# Frequency of Trying to Lose Weight and Its Association with Children’s Weight Perception and Dietary Intake (NHANES 2011–2012)

**DOI:** 10.3390/nu11112703

**Published:** 2019-11-08

**Authors:** Taiya Brown, Francine Overcash, Marla Reicks

**Affiliations:** Department of Food Science and Nutrition, University of Minnesota, 1334 Eckles Avenue, St. Paul, MN 55108, USA; brow4102@umn.edu (T.B.); overc006@umn.edu (F.O.)

**Keywords:** child, adolescent, dieting, weight perception, macronutrients, micronutrients, food groups

## Abstract

Weight loss practices and frequency among children and adolescents can impact overall diet quality. We used cross-sectional U.S. National Health and Nutrition Examination Survey data (NHANES) 2011–2012 from 1303 children and adolescents (8–15 years) to examine associations between frequency of trying to lose weight (never vs. sometimes/a lot) and sociodemographic characteristics, self-perception of weight, and dietary intake. A greater frequency of trying to lose weight was reported by participants with overweight/obesity, those from households with lower annual income and those who perceived they were overweight or obese compared to their counterparts. A high proportion of participants with overweight and obesity considered themselves to be “about the right weight” (76.7 and 42.8%, respectively). Intake data based on one 24-h dietary recall were examined using multivariable regression models adjusted for child and parent/family characteristics. In adjusted analyses, energy and total fat intakes were lower among those trying to lose weight compared to those never trying to lose weight. Intakes of cholesterol, sodium, and refined grains were not different by frequency of trying to lose weight (all *p* = 0.059–0.074). Weight loss efforts may be related to better nutritional profiles for some children and adolescents.

## 1. Introduction

Children and adolescents use weight control practices based on body weight perception [1,2,3,4] and peer, media, and parental influences [5,6,7]. Among a nationally representative sample of United States (U.S.) children and adolescents based on National Health and Nutrition Examination Survey data (NHANES 2005–2010), frequency of trying to lose weight was commonly reported by the majority of those who perceived themselves as overweight even when the perception was inaccurate (93% of girls and 68% of boys 8–11 years, 91% of girls and 76% of boys 12–15 years) [4]. Weight control methods may range from healthy behaviors including cutting back on portion sizes and sweets/snacks and participating in regular family meals, to extremely unhealthy practices such as diet pill and laxative usage, vomiting, and skipping meals [8]. Self-reported, unhealthy dieting behaviors among U.S. adolescents were more likely among individuals who misperceived their weight status based on Youth Risk Behavior Surveillance System data (1999–2013) compared to a reference group with normal weight and an accurate perception of their weight status [9]. Unhealthy versus healthy weight management behaviors are likely to negatively impact diet quality.

Limited studies have reported relationships between trying to lose weight and dietary intake or quality among children and adolescents. Positive associations were observed between trying to lose weight and greater fruit and vegetable consumption among girls [10] and all adolescents [11] and lower sugar, sodium, and fat intakes [12]. Boys (10–15 years) who reported trying to lose weight, (NHANES 2005–2014) had lower calorie intake and percent calories from fat [13]. Associations were also observed between unhealthy practices and lower intakes of fruits, vegetables, fiber, calcium, and iron [14,15].

Late childhood and early adolescence are critical periods for physical growth and psychosocial development. Weight gain typically occurs as part of healthy physical development during puberty [16]; but may trigger unhealthy dieting behaviors in some children and adolescents [17]. Therefore, late childhood and early adolescence represents a window of opportunity to promote healthy eating behaviors that can be maintained into mid and late adolescence and early adulthood.

The relationship between trying to lose weight, food, and nutrient intakes needs to be examined further among older children and adolescents based on the negative consequences reported for unhealthy dieting behaviors, which may coincide with weight misperceptions. Therefore, the purpose of this study was to determine differences in demographic characteristics, self-perception of weight, and food group and nutrient intakes of children (8–11 years) and adolescents (12–15 years) by report of trying to lose weight (never versus sometimes/a lot) using a nationally representative U.S. sample of children and adolescents. In addition, a secondary purpose was to assess the frequency of weight loss methods among those who reported trying to lose weight.

## 2. Materials and Methods 

### 2.1. Participants

The study sample included participants aged 8–15 years from the National Health and Nutrition Examination Survey (NHANES) 2011–2012 [18]. Personal interview data collected from participants 8–15 years at the Mobile Examination Center (MEC) included weight history information including frequency of weight loss efforts, self-perception of weight, reasons for losing weight, and methods used to lose weight. Later NHANES data collection cycles collected this information only from adolescents ≥16 years, but not from those 8–15 years. Data collection details were included in the NHANES 2011–2012 Procedure Manuals available at the Centers for Disease Control and Prevention (CDCP), National Center for Health Statistics (NCHS) NHANES website [19]. For this study, participants were included if they had measured weight and height, and information on self-perception of weight, weight loss efforts and methods (*n* = 1303). The NCHS Institutional Review Board for the Ethics Review Board approved NHANES data collection (Protocol #2011-17) [20]. Written informed consent was obtained from participants before data collection. The University of Minnesota Institutional Review Board determined that the analysis completed for the current study was not research involving human subjects based on the use of de-identified, publicly available data.

### 2.2. Sociodemographic Variables

Family and youth sociodemographic information was collected in the home by trained interviewers including sex, age, ethnicity (non-Hispanic White, non-Hispanic Black, Mexican American and Hispanic, and other), family income level, and the education level of the household reference person. Annual family income level was determined by a ratio of family income to poverty threshold and was recoded into two levels (≤$ 34,999, ≥$ 35,000). Level of education for the household reference person was collapsed from eight into two levels for analysis (High school grad/General Equivalency Diploma or equivalent, some college/college grad/graduate school). Age was also dichotomized into two levels (<11 years, ≥11 years). 

### 2.3. Body Mass Index

Height and weight were measured in the MEC by trained health professionals using standardized procedures [21]. BMI was calculated based on measured height and weight (kg/m^2^) and converted to BMI-percentile for age and sex using a Statistical Analysis System (SAS) program (CDCP sex-specific 2000 BMI-for-age growth charts for the U.S.) [22]. BMI-percentile was categorized according to four groups: underweight BMI <5th percentile, normal weight—BMI 5th to <85th percentiles, overweight—BMI 85th to <95th percentiles, and obese—BMI ≥ 95th percentiles. For some analyses, BMI-percentile was collapsed into two groups (underweight and normal, overweight and obese). 

### 2.4. Self-Perception of Weight

Participants were asked about their perception of their weight with the question: “Do you consider yourself now to be…?” with three response options: (fat or overweight, too thin, about the right weight). Proxy respondents were not used for this assessment. A variable was created for further analysis based on whether the respondents accurately perceived their weight according to their measured weight (accurate perceivers vs. inaccurate perceivers).

### 2.5. Weight Loss Efforts and Methods

Participants were asked “In the past year, how often have you tried to lose weight? Would you say…” Response options were never, sometimes, or a lot. Frequency of trying to lose weight was dichotomized as never vs. sometimes and a lot. Proxy respondents were not used for this assessment. For those who responded sometimes or a lot, additional questions regarding various types of weight loss methods were included such as dieting, starving, cutting back on what was eaten, skipping meals, exercising, and eating less sweets or fatty foods. Participants were asked “In the past year, how often have you (e.g., been on a diet) to lose weight? Would you say…” Response options were never, sometimes, or a lot.

### 2.6. Self-Reported Dietary Intake

The 24-h dietary recall data for the first day were used to report intake from foods and beverages for energy, macronutrients, total sugars, dietary fiber, and selected micronutrients [23]. The first dietary recall was collected in person in the MEC. Dietary recall interviews for participants age eight years old were conducted with a proxy adult with the child present. Interviews for participants aged 9–11 were conducted with the child present along with an adult familiar with the participant’s intake to assist in the recall process. Participants aged 12–15 answered for themselves and no proxies or adults were present. The dietary interviews were conducted in Spanish or English with translators available if needed. Information from the Food Patterns Equivalents Databases (FPED) for 2011–2012 was used to report food group information [24].

### 2.7. Data Analysis

Data analyses were conducted using SAS^®^ (SAS Institute Inc., Cary, NC, USA, version 9.4) Survey Procedures (e.g., surveyfreq, surveymeans, surveyreg) to account for NHANES’s complex, multistate, probability sampling design to ensure representativeness of the civilian, noninstitutionalized U.S. population. Appropriate sampling weights were applied to account for the complex survey design. Comparisons for demographic, anthropometric, and weight perception variables based on the weight loss effort frequency question (Never vs. Sometimes/A Lot) were performed using chi-square tests (proc surveyfreq). The same SAS procedure was performed to determine if weight perception (accurate vs. nonaccurate) differed by BMI-percentile category. Means and standard errors of the dietary intake variables by weight loss effort were calculated and compared using t-tests (proc surveymeans). Further comparisons were made using least square means and standard errors of dietary intake variables by weight loss frequency effort using multivariable regression models (proc surveyreg) that adjusted for age, race, BMI status, and annual household income. Among those who reported trying to lose weight sometimes/a lot, frequency of various weight loss methods used was determined.

## 3. Results

### 3.1. Weight Loss Efforts by Demographic Characteristics, Weight Status, and Body Weight Perception 

About half of the sample (53.9%) reported trying to lose weight sometimes (*n* = 548, 42.1%) or a lot (*n* = 154, 11.8%) in the past year. No differences were observed in the frequency of trying to lose weight by age, sex, or education level of the household reference person (Table 1). Participants from households with an annual income ≤$34,999 reported a higher frequency of trying to lose weight compared to participants living in homes with greater annual family income. Participants with overweight or obesity were more likely to report trying to lose weight, compared to participants having an underweight or normal weight status (*p* < 0.0001). The results showed that 91.7% of the participants who perceived themselves to be “fat or overweight” reported trying to lose weight sometimes/a lot compared to less than half of participants who perceived themselves to be “about the right weight” (48.4%) or “too thin” (23.2%) (*p* < 0.0001). For participants who accurately perceived their weight status, 52.4% tried to lose weight sometimes/a lot, while 47.6% never tried to lose weight (*p* = 0.006). Inaccurate weight perception was more often observed among participants who tried to lose weight sometimes/a lot versus those who never tried to lose weight. Race/ethnicity differences were observed for frequency of trying to lose weight. The proportion of non-Hispanic Blacks who reported trying to lose weight sometimes/ a lot (54.7%) was significantly greater than Non-Hispanic Whites (49.7%) and Others (47.9%) (*p* = 0.004). 

### 3.2. Actual Weight and Self-Perception of Weight 

Among the total number of participants, 3.3%, 58.9%, 15.8%, and 21.9% had underweight, normal weight, overweight, and obese weight status, respectively, by actual BMI-percentile (Table 2). Of those with underweight, 37.2% considered themselves to be too thin and 62.8% considered themselves to be normal weight. For participants with normal weight, 85.2% considered themselves to be about the right weight, 11.1% considered themselves to be too thin and 3.7% considered themselves to be fat or overweight. For the participants with overweight, 21.4% accurately perceived themselves to have overweight, whereas 76.7% considered themselves to be about the right weight and 1.9% considered themselves to be too thin. Of the participants with obesity, 54.7% accurately perceived themselves as having overweight, 42.8% perceived themselves to be of normal weight, and 2.1% perceived themselves as too thin.

### 3.3. Food Group and Nutrient Intake by Frequency of Weight Loss Effort

In the unadjusted analysis, intakes of energy, carbohydrates, total sugars, fat, cholesterol, sodium, and refined and whole grains were lower among those who reported trying to lose weight versus never trying (Table 3). However, adjusted models only showed that energy and total fat intakes were significantly lower for participants who reported trying to lose weight compared to those who reported never trying to lose weight. Fruit, vegetable, and whole grain food intakes were not different by frequency of trying to lose weight in the adjusted models. Adjusted models for differences in intakes of nutrients and food groups of concern including cholesterol, sodium, and refined grains were nonsignificant (*p*-values from 0.059 to 0.074).

### 3.4. Weight Loss Methods

For those who reported trying to lose weight (*n* = 702), methods to lose weight included exercising (*n* = 698, 99.4%) eating less sweets or fatty foods (*n* = 623, 88.7%), cutting back on what was eaten (*n* = 523, 74.5%), dieting (*n* = 328, 46.7%), skipping meals (*n* = 318, 45.3%), and starving for a day or more (*n* = 162, 23.1%).

## 4. Discussion

This study examined sociodemographic characteristics, self-perception of weight, and food group and nutrient intakes associated with trying to lose weight among a large U.S. nationally representative sample of children and adolescents (8–15 years). Results showed that the frequency of trying to lose weight varied by sociodemographic characteristics, a high percentage of participants with overweight or obesity considered themselves to be about the right weight (77% and 43%, respectively), and trying to lose weight was associated with lower energy and fat intakes.

Sociodemographic factors, such as age, sex, and family income may be related to the frequency of trying to lose weight; however, the relationship has been variable for some factors between NHANES studies. For example, an NCHS data brief (NHANES 2013–2016) reporting results from older adolescents (16–19 years) [25] indicated that 38% reported trying to lose weight, while in the current study, 54% of participants (8–15 years) reported trying to lose weight. Irish children and adolescents (9–18 years) participating in focus group interviews perceived that parents had less control over food choices of older adolescents versus younger adolescents and children [26]. Therefore, greater autonomy over food choices and responsibility for food acquisition and preparation among older adolescents may result in a different conceptualization of dieting versus younger children and adolescents, which could influence their reports of frequency of trying to lose weight. Among older adolescents (16–19 years, NHANES 2013–2016), 45% of girls compared to 30% of boys reported trying to lose weight, whereas in the current study, 56% of girls compared to 52% of boys (8–15 years) reported trying to lose weight. Gender differences in body image ideals may not be as apparent among younger children and adolescents compared to older adolescents. These differences could be based in part on the relationship between the development of body image and age-related physical transitions, which may occur at different ages among girls versus boys [27]. In addition, in the current study, those from households with lower annual income reported a higher frequency of trying to lose weight compared to those from households with higher income. A meta-analysis of three datasets including U.S. nationally representative data (1971–2014) showed that children and adolescents from low-income households had an increased risk of obesity compared to children and adolescents in middle and high-income households [28]. These findings indicate that efforts to lose weight may be more frequent among lower versus higher income children and adolescents because of a higher incidence of overweight and obesity. 

The current study showed that inaccurate perception of weight status was prevalent among 8–15-year-olds with over one-third incorrectly self-assessing their weight status compared to their actual BMI status. Among those with overweight and obesity, 77% and 43%, respectively, considered themselves to be about the right weight. Nationally representative U.S. data from the Early Childhood Longitudinal Study (2006–2007) also showed that a majority of adolescents with overweight and obesity perceived their weight status as about right or slightly overweight, respectively [29]. Data from 1999–2013 regarding the proportion of U.S. adolescents with overweight and obesity who misperceived their weight status as normal weight showed no linear temporal trends [30] consistent with findings based on data collected in 2006–2007 [29] and the current study. Misperception of overweight status among adults and adolescents has been attributed to sociocultural influences including social weight comparisons [31,32]. NHANES data from adults (1988–1994 and 1999–2004) showed a shift in social norms over time regarding the self-perception of overweight status as the prevalence of adult and childhood obesity increased [33]. The same shift may have occurred among children and adolescents as the prevalence of childhood obesity increased over time. In the current study, those with inaccurate versus accurate weight perceptions were more likely to report trying to lose weight (58% vs. 52%). NHANES data from 2005–2010 [4] showed a high frequency of trying to lose weight for those with both inaccurate and accurate perceived overweight (8–15 years). Inaccurate perceptions of weight status may lead to discrepancies between actual dietary intake and optimal intake. For example, weight loss attempts by adolescents with normal weight status may result in unnecessary dietary restrictions, which could result in nutrient deficiencies and lower diet quality. Because body image concerns, body satisfaction, and eating behaviors of children and adolescents are influenced by social, cultural, and family factors, misperceptions regarding body weight and altered eating patterns could be addressed at multiple levels via a variety of delivery channels.

The finding that total energy (kcal) and total fat intakes were lower among adolescents who reported trying to lose weight compared to those who reported never trying to lose weight was in the expected direction given previous studies. Generally, adolescents who dieted made healthier food choices that resulted in lower sugar [12], lower fat [12], and higher fiber intakes [34]. A study by James et al. [35] involving college students found that those trying to lose weight avoided carbohydrates more often than those not trying to lose weight. Among participants in NHANES (2005–2104), boys (10–15 years) but not girls who reported trying to lose weight consumed fewer calories and had a lower percentage of calories from fat [13]. About 89% of participants in the current study reported eating less sweets or fatty foods as a method to lose weight, which might have contributed to the lower energy and fat intakes observed among all participants. Reduction of energy intake and sweets and fatty foods is in line with current public health recommendations regarding healthy weight control behaviors for adolescents based on avoidance of calorie-dense, nutrient-poor foods [36]. Adolescents who try to lose weight may be eating fewer fatty and sweet foods based on knowledge about healthy food choices acquired from parents, school health classes, food product labeling, and web-based information. 

In the current study, children and adolescents reported using healthy weight loss methods such as exercising and eating fewer sweets or fatty foods more often than unhealthy methods. Similar results were obtained from nationally representative samples of adolescents from >30 countries [37] indicating that exercising and eating fewer sweets were the most commonly used practices among adolescents. However, the prevalence of these behaviors varied somewhat by weight status and sex. A study of trends in prevalence of weight loss behaviors showed that exercise as a weight management behavior increased in prevalence among boys (9th–12th grade) from 1999 to 2009 in the U.S., while the prevalence among girls did not change [38]. In addition, among middle school children, regardless of sex or dieting status, those who were exercising to manage weight were more likely to eat for hunger instead of emotional response [39]. Exercise should continue to be encouraged as a weight management behavior with opportunities made widely available for both boys and girls. Exercise should be promoted especially for low-income youth based on results from the current study, which showed that more youth from lower income households were likely to try to lose weight than those from higher income households.

A strength of this study was the use of a large, nationally representative sample of children and adolescents. Limitations include the use of cross-sectional data, which does not allow for demonstration of cause and effect relationships. Other limitations include use of a single dietary recall and the inherent issues regarding possible under or over reporting of dietary intake based on difficulty with recall or social desirability bias. One study found differential misreporting among subgroups of children and adolescents using NHANES data [40]. Under-reporting was more prevalent among the oldest age group (12–19 years) compared to the younger age groups (≤5 and 6–11 years), non-Hispanic Blacks vs. non-Hispanic whites, and participants with overweight/obesity vs. normal weight. Therefore, the findings from the current study should be interpreted with caution. Parent-assisted or proxy reports of 24-h dietary intake of youth are subject to social desirability bias. However, the only known unbiased method of energy intake, doubly labelled water, is impractical for large-scale epidemiological studies. The USDA’s Automated Multiple-Pass Method remains the recommended method for quantifying intakes with the least bias for large, population-level dietary surveys [41]. In addition, weight misperception was assessed with only one question regarding consideration of weight status.

The findings from the current study may be used to guide public health policies, future interventions and research. Public health policies to reduce weight-based bullying and discrimination within schools and other public settings are necessary to promote positive body image and satisfaction, which may affect the prevalence of weight misperceptions and possible unhealthy dieting behaviors. Physicians and school personnel could focus on helping youth and families accurately interpret the results of weight measurements done in clinics or schools. Intervention programs could include a continued emphasis on accurate perception of weight status based on a recognition that body image and satisfaction, and eating behaviors are influenced by individual, family, and societal factors. Therefore, these issues need to be addressed at multiple venues (home, school, community, and clinical settings) via a variety of channels (in-person programs, online media). Future research could include further characterization of age, sex, and income differences in frequency of trying to lose weight based on findings from the current study and others [4,25]. Research could also be conducted to determine the specific dietary changes that account for differences in energy and total fat intake by frequency of trying to lose weight regarding types of foods, eating occasions, and situational context. 

In summary, demographic and physical characteristics were related to trying to lose weight among children and adolescents including race/ethnicity, family income, and weight status. A substantial number of youth had inaccurate perceptions of their weight status. Results adjusted for covariates showed that intake of energy and total fat were lower among those trying to lose weight sometimes or a lot compared to never trying to lose weight. Exercise and eating fewer sweets or fatty foods were common weight loss methods. Nutritional profiles may be better for some children and adolescents who report trying to lose weight.

## Figures and Tables

**Table 1:** Demographic and physical characteristics and self-perception of weight by how often tried to lose weight ^1^.

		Tried to Lose Weight ^2^		
	All *n* = 1303 *n* (%)	Sometimes/A Lot *n* = 702 *n* (%)	Never *n* = 601 *n* (%)	*p*-Value ^3^
Age (*n* = 1303)				0.911
<11 years	729 (55.9)	400 (54.9)	329 (45.1)	
≥11 years	574 (44.1)	302 (52.6)	272 (47.4)	
Sex (*n* = 1303)				0.055
Boy	661 (50.7)	343 (51.9)	318 (48.1)	
Girl	642 (49.3)	359 (55.9)	283 (44.1)	
Race/ethnicity (*n* = 1303)				0.004
Mexican-American/Hispanic	403 (30.9)	204 (50.6)	163 (40.4)	
Other	217 (16.7)	104 (47.9)	113 (52.1)	
Non-Hispanic White	310 (23.8)	154 (49.7)	156 (50.3)	
Non-Hispanic Black	373 (28.6)	204 (54.7)	169 (45.3)	
Annual family income (*n* = 1228)				0.017
≤34,999	550 (44.8)	319 (58.0)	231 (42.0)	
≥35,000	678 (55.2)	338 (49.9)	340 (50.1)	
Education level household reference person (*n* = 1262)				0.112
High school grad/GED ^4^	608 (48.2)	362 (59.5)	246 (40.5)	
Some college/college	654 (51.8)	333 (50.9)	321 (49.1)	
Weight status ^5^ (*n* = 1254)				<0.0001
Underweight/normal	765 (61.0)	297 (38.8)	468 (61.2)	
Overweight/obese	489 (39.0)	400 (81.8)	89 (18.2)	
How do you consider your weight? (*n* = 1302)				<0.0001
Fat or overweight	228 (17.5)	209 (91.7)	19 (8.3)	
Too thin	112 (8.6)	26 (23.2)	86 (76.8)	
About the right weight	962 (73.9)	466 (48.4)	496 (51.6)	
Perception of weight status ^6^ (*n* = 1296)				0.006
Accurate	869 (67.1)	455 (52.4)	414 (47.6)	
Inaccurate	427 (32.9)	246 (57.6)	181 (42.4)	

^1^ Column percent for All and row percent for How Often Tried to Lose Weight category. ^2^ In the past year, how often have you tried to lose weight? Would you say…” (never, sometimes, or a lot). ^3^ *p*-value according to Chi-square tests (significance level = 0.05). ^4^ GED–General Equivalency Diploma. ^5^ BMI-percentiles-Center for Disease Control and Prevention sex-specific 2000 BMI-for-age U.S. growth charts and weight categories. ^6^ Accurate = perception matches actual BMI category, inaccurate = perception does not match actual BMI category.

**Table 2:** Accuracy of weight perception by actual BMI-percentile category ^1^ (*n* = 1300).

	BMI status ^3^			
Weight Perception ^2^	Underweight *n* (%)	Normal *n* (%)	Overweight *n* (%)	Obese *n* (%)
Too thin	16 (37.2)	85 (11.1)	4 (1.9)	6 (2.1)
About the right weight	27 (62.8)	653 (85.2)	158 (76.7)	122 (42.8)
Fat or overweight	0 (0)	28 (3.7)	44 (21.4)	156 (54.7)
Total	43 (3.3)	766 (58.9)	206 (15.8)	285 (21.9)

^1^ Column percent for weight perception by BMI status. ^2^ Do you consider yourself now to be…? (fat or overweight, too thin, or about the right weight). ^3^ Based on BMI-percentiles-Center for Disease Control and Prevention sex-specific 2000 BMI-for-age U.S. growth charts and weight categories.

**Table 3:** Food group and nutrient intakes by how often tried to lose weight ^1.^

Nutrient	All Mean (SE) *n* = 1303	How Often Tried to Lose Weight ^2^					
		Non-Adjusted			Adjusted Models ^3^		
		Sometimes, a Lot Mean (SE) *n* = 702	Never Mean (SE) *n* = 601	*p*-Value ^4^	Sometimes/a Lot LS Mean (SE) *n* = 702	Never LS Mean (SE) *n* = 601	*p*-Value ^5^
Energy (calories)	2005 (35)	1928 (55)	2089 (29)	0.009	1914 (59)	2073 (30)	0.035
Protein (g)	71 (2)	69 (3)	73 (2)	0.194	68 (3)	73 (1)	0.156
Carbohydrate (g)	270 (4)	260 (6)	280 (4)	0.010	260 (7)	275 (5)	0.108
Total sugars (g)	130 (3)	125 (3)	135 (4)	0.048	127 (4)	132 (4)	0.307
Fiber (g)	14.7 (0.3)	14.4 (0.4)	14.9 (0.5)	0.235	14.2 (0.5)	14.7 (0.5)	0.490
Fat (g)	74 (2)	71 (3)	78 (2)	0.014	70 (3)	78 (2)	0.037
Cholesterol (mg)	225 (8)	213 (15)	240 (11)	0.030	204 (14)	243 (11)	0.061
Calcium (mg)	1080 (22)	1077 (28)	1084 (41)	0.599	1053 (26)	1093 (40)	0.367
Iron (mg)	14.8 (0.5)	14.3 (0.6)	15.4 (0.7)	0.074	14.2 (0.7)	15.0 (0.6)	0.383
Sodium (mg)	3207 (79)	3099 (114)	3319 (86)	0.049	3070 (124)	3324 (74)	0.059
Potassium (mg)	2279 (34)	2229 (66)	2326 (63)	0.228	2193 (61)	2323 (59)	0.182
Food group							
Total fruit (svg)	1.1 (0.1)	1.2 (0.1)	1.0 (0.1)	0.527	1.2 (0.1)	1.0 (0.1)	0.120
Fruit juice (cup eq)	0.4 (0.0)	0.4 (0.0)	0.3 (0.0)	0.901	0.3 (0.0)	0.3 (0.0)	0.754
Total vegetable (svg)	0.9 (0.0)	0.9 (0.1)	0.9 (0.1)	0.322	0.9 (0.1)	0.9 (0.1)	0.671
Potatoes (cup eq)	0.3 (0.0)	0.3 (0.0)	0.3 (0.0)	0.106	0.3 (0.0)	0.3 (0.0)	0.562
Added sugars (g)	20.0 (0.7)	18.9 (0.8)	21.1 (0.8)	0.057	19.1 (0.9)	20.7 (0.8)	0.209
Refined grains (svg)	6.4 (0.2)	6.1 (0.2)	6.6 (0.2)	0.024	6.0 (0.3)	6.6 (0.2)	0.074
Whole grains (cup eq)	0.7 (0.0)	0.7 (0.1)	0.8 (0.1)	0.044	0.7 (0.1)	0.8 (0.1)	0.285

^1^ SE = standard error of the means (variance/square root of N); LS Mean = least squares mean; svg = daily servings; cup eq = cup equivalents. ^2^ In the past year, how often have you tried to lose weight? Would you say…” (never, sometimes, or a lot). ^3^ Adjusted for covariates: age, race/ethnicity, BMI status, and annual household income. ^4^ *p*-value according to *t*-tests; (significance level = 0.05). ^5^ *p*-value according to multivariate regression models; (significance level = 0.05).
